# The Relevance of Advanced Therapy Medicinal Products in the Field of Transplantation and the Need for Academic Research Access: Overcoming Bottlenecks and Claiming a New Time

**DOI:** 10.3389/ti.2023.11633

**Published:** 2023-09-27

**Authors:** Lorenzo Piemonti, Hanne Scholz, Dide de Jongh, Julie Kerr-Conte, Aart van Apeldoorn, James A. M. Shaw, Marten A. Engelse, Eline Bunnik, Markus Mühlemann, Karolina Pal-Kutas, William E. Scott, Jérémy Magalon, Patrick Kugelmeier, Ekaterine Berishvili

**Affiliations:** ^1^ Diabetes Research Institute, IRCCS Ospedale San Raffaele and Vita-Salute San Raffaele University, Milan, Italy; ^2^ Department of Transplant Medicine and Institute for Surgical Research, Oslo University Hospital, Oslo, Norway; ^3^ Department of Medical Ethics, Philosophy and History of Medicine, Erasmus MC, University Medical Centre Rotterdam, Rotterdam, Netherlands; ^4^ Department of Nephrology and Transplantation, Erasmus MC, University Medical Centre Rotterdam, Rotterdam, Netherlands; ^5^ Université de Lille, INSERM, Campus Hospitalo-Universitaire de Lille, Institut Pasteur de Lille, U1190-EGID, Lille, France; ^6^ Department CBITE, MERLN Institute for Technology-Inspired Regenerative Medicine, Maastricht University, Maastricht, Netherlands; ^7^ Translational and Clinical Research Institute, The Medical School, Newcastle University, Newcastle upon Tyne, United Kingdom; ^8^ Nephrology, Leiden University Medical Center, Leiden, Netherlands; ^9^ Kugelmeiers Ltd., Zollikerberg, Switzerland; ^10^ Laboratoire de Culture et Thérapie Cellulaire, Assistance Publique des Hôpitaux de Marseille, Marseille, France; ^11^ Vascular Research Center Marseille, INSERM UMRS 1076, Faculté de Pharmacie, Marseille, France; ^12^ Laboratory of Tissue Engineering and Organ Regeneration, Department of Surgery, University of Geneva, Geneva, Switzerland

**Keywords:** advanced therapy medicinal products (ATMPs), regulatory processes, clinical trials, rare diseases, transplantation

## Abstract

The field of transplantation has witnessed the emergence of Advanced Therapy Medicinal Products (ATMPs) as highly promising solutions to address the challenges associated with organ and tissue transplantation. ATMPs encompass gene therapy, cell therapy, and tissue-engineered products, hold immense potential for breakthroughs in overcoming the obstacles of rejection and the limited availability of donor organs. However, the development and academic research access to ATMPs face significant bottlenecks that hinder progress. This opinion paper emphasizes the importance of addressing bottlenecks in the development and academic research access to ATMPs by implementing several key strategies. These include the establishment of streamlined regulatory processes, securing increased funding for ATMP research, fostering collaborations and partnerships, setting up centralized ATMP facilities, and actively engaging with patient groups. Advocacy at the policy level is essential to provide support for the development and accessibility of ATMPs, thereby driving advancements in transplantation and enhancing patient outcomes. By adopting these strategies, the field of transplantation can pave the way for the introduction of innovative and efficacious ATMP therapies, while simultaneously fostering a nurturing environment for academic research.

Advanced Therapy Medicinal Products (ATMPs) defined in Regulation (EC) No 1394/2007 in the European Union, are medicinal products for human use including gene therapy medicinal products (GTMP), somatic cell therapy medicinal products (sCTMP), tissue-engineered products (TEP), or combinations of these [1]. The European Medicines Agency (EMA) regulates ATMPs through the Committee for Advanced Therapies (CAT), which provides scientific advice and evaluates marketing authorization applications for ATMPs based on quality, safety, and efficacy. The CAT’s opinion forms the basis for marketing authorization by the European Commission. As of the most recent published report (Figure 1, quarterly highlights and approved ATMPs, 2009-January 2023), the CAT provided 597 scientific recommendations on ATMPs, 559 scientific advice to companies, and reviewed 116 applications for Priority Medicines designation, granting 50 of them. The first ATMP to receive authorization in the EU was ChondroCelect®, a tissue-engineered product used for treating cartilage defects in 2009, followed by Glybera®, the first gene therapy, in 2012, and PROVENGE®, the first somatic cell therapy, in 2013.

**FIGURE 1:**
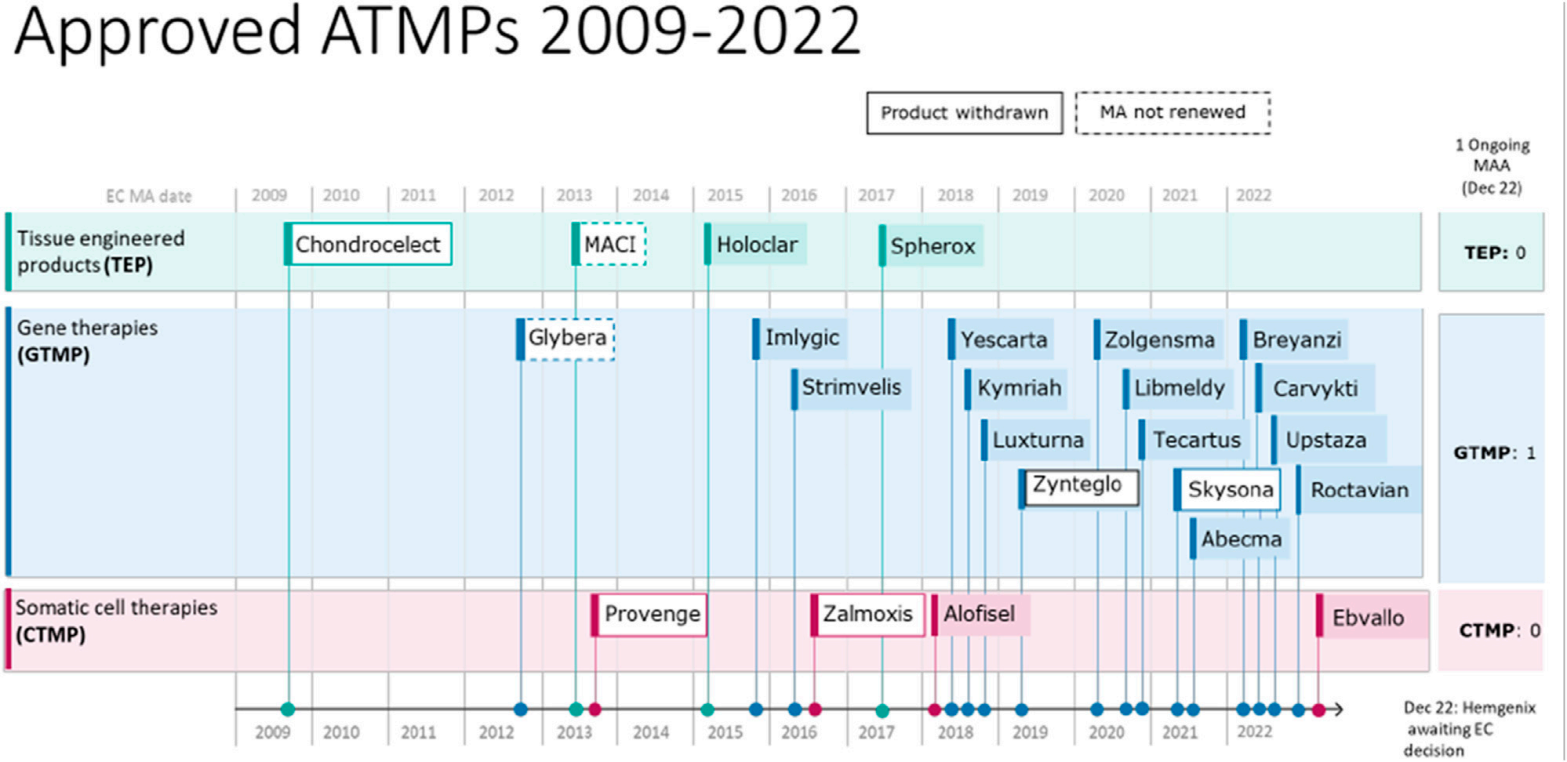
Summary of authorized Advanced Therapy Medicinal Products (ATMPs) between 2009 and 2022, encompassing both withdrawn or non-renewed ones. Source: [2].

The field of ATMPs is still relatively new, and the number of Marketing Authorization Applications (MAAs) submitted to CAT remains low [3]. Despite the high level of presubmission activity, the CAT only received 36 MAAs for ATMPs and authorized 25 of them (Table 1). This is partly due to the complex and challenging nature of developing ATMPs, which often requires a significant investment of time and resources to achieve regulatory approval [4, 5]. One reason for the slow take/emergence of ATMPs is the limited patient populations for rare diseases, which makes it challenging to conduct clinical trials and demonstrate safety and efficacy [6]. Another factor is the evolving regulatory landscape, which requires the establishment of clear guidelines and standards for the development and approval of these therapies. Additionally, ATMPs are often developed for diseases with limited treatment options, which can pose additional challenges for clinical trial design and regulatory approval (i.e., small patient populations, disease heterogeneity, lack of established endpoints, limited comparative data, long-term follow-up requirements, regulatory complexities, and specialized manufacturing).

**TABLE 1:** List of authorised ATMPs by EMA.

NAME	INN	Active substance	Type of ATMP	Indication	Company	Authorisation Date	Orphan	PRIME	MA
Chondrocelect		Characterized viable autologous cartilage cells expanded *ex vivo* expressing specific marker proteins	TEP	Repair of single symptomatic cartilage defects of the femoral condyle of the knee in adults	TiGenix N.V.	5/10/2009	No	No	withdrawn July 2016
Glybera	alipogene tiparvovec	human lipoprotein lipase (LPL) gene variant LPLS447X in a vector. The vector comprises a protein shell derived from adeno-associated virus serotype 1, the Cytomegalovirus promoter, a woodchuck hepatitis virus posttranscriptional regulatory element and AAV2 derived inverted terminal repeats	GTMP	Familial lipoprotein lipase deficiency (LPLD)	uniQure biopharma B.V.	25/10/2012	Yes	No	not renewed; ended Oct. 2017
MACI		Autologous cultured chondrocytes	TEP, combined ATMP	Repair of symptomatic cartilage defects of the knee	Vericel Denmark ApS	27/06/2013	No	No	not renewed; ended June 2018
Provenge	Sipuleucel-T	Autologous peripheral-blood mononuclear cells including a minimum of 50 million autologous CD54^+^ cells activated with prostatic acid phosphatase granulocyte-macrophage colony-stimulating factor	CTMP	Treatment of asymptomatic or minimally symptomatic metastatic (non-visceral) castrate-resistant prostate cancer in male adults in whom chemotherapy is not yet clinically indicated	Dendreon UK Ltd	6/09/2013	No	No	withdrawn May 2015
Holoclar		*Ex vivo* expanded autologous human corneal epithelial cells containing stem cells	TEP	Treatment of adult patients with moderate to severe limbal stem cell deficiency, unilateral or bilateral, due to physical or chemical ocular burns	Holostem Terapie Avanzate s.r.l	17/02/2015	Yes	No	
Imlygic	talimogene laherparepvec	Attenuated herpes simplex virus type-1 (HSV-1) derived by functional deletion of 2 genes (ICP34.5 and ICP47) and insertion of coding sequence for human granulocyte macrophage colony-stimulating factor (GM-CSF)	GTMP	Unresectable melanoma that is regionally or distantly metastatic	Amgen Europe B.V.	16/12/2015	No	No	
Strimvelis		Autologous CD34^+^ enriched cell fraction that contains CD34^+^ cells transduced with retroviral vector that encodes for the human adenosine deaminase (ADA) cDNA sequence from human haematopoietic stem/progenitor (CD34^+^) cells	GTMP	Severe combined immunodeficiency due to adenosine deaminase deficiency (ADA-SCID)	Orchard Therapeutics (Netherlands) BV	26/05/2016	Yes	No	
Zalmoxis		Allogeneic T cells genetically modified with a retroviral vector encoding for a truncated form of the human low affinity nerve growth factor receptor and the herpes simplex I virus thymidine kinase	CTMP	Adjunctive treatment in haploidentical haematopoietic stem cell transplantation (HSCT) of adult patients with high-risk haematological malignancies	MolMed SpA	18/08/2016	Yes	No	withdrawn Oct. 2019
Spherox		Spheroids of human autologous matrix-associated chondrocytes	TEP	Repair of symptomatic articular cartilage defects of the femoral condyle and the patella of the knee	CO.DON Gmbh	10/07/2017	No	No	
Alofisel	Darvadstrocel	Expanded human allogeneic mesenchymal adult stem cells extracted from adipose tissue	CTMP	Treatment of complex perianal fistulas in adult patients with non-active/mildly active luminal Crohn’s disease, when fistulas have shown an inadequate response to at least one conventional or biologic therapy	Takeda Pharma A/S	23/03/2018	Yes	No	
Yescarta	Axicabtagene ciloleucel	Autologous T cells transduced *ex vivo* using a retroviral vector expressing an anti-CD19 chimeric antigen receptor (CAR) comprising a murine anti-CD19 single chain variable fragment linked to CD28 co-stimulatory domain and CD3-zeta signalling domain	GTMP	Diffuse large B cell lymphoma (DLBCL) and high-grade B-cell lymphoma (HGBL)	Kite Pharma EU B.V.	23/08/2018	Yes	Yes	
Kymriah	Tisagenlecleucel	Autologous T cells genetically modified *ex vivo* using a lentiviral vector encoding an anti-CD19 chimeric antigen receptor (CAR)	GTMP	B cell acute lymphoblastic leukaemia (ALL); diffuse large B cell lymphoma (DLBCL); follicular lymphoma (FL)	Novartis Europharm Limited	23/08/2018	Yes	Yes	
Luxturna	voretigene neparvovec	Gene transfer vector that employs an adeno-associated viral vector serotype 2 capsid as a delivery vehicle for the human retinal pigment epithelium 65 kDa protein (hRPE65) cDNA to the retina	GTMP	Inherited retinal dystrophy caused by confirmed bi-allelic RPE65 mutations	Novartis Europharm Limited	22/11/2018	Yes	No	
Zynteglo	Betibeglogene autotemcel	Genetically modified autologous CD34^+^ cell enriched population that contains haematopoietic stem cells transduced with lentiviral vector encoding the βA-T87Q-globin gene	GTMP	Transfusion-dependent β thalassaemia	Bluebird bio (Netherlands) B.V.	29/05/2019	Yes	Yes	withdrawn March 2022
Zolgensma	Onasemnogene abeparvovec	Non-replicating recombinant adeno-associated virus serotype 9 based vector containing the cDNA of the human SMN gene under the control of the cytomegalovirus enhancer/chicken-β-actin-hybrid promoter	GTMP	Spinal muscular atrophy (SMA)	Novartis Europharm Limited	18/05/2020	Yes	Yes	
Libmeldy	Atidarsagene autotemcel	Genetically modified autologous CD34^+^ cells enriched population that contains haematopoietic stem and progenitor cells (HSPC) transduced *ex vivo* using a lentiviral vector expressing the human arylsulfatase A (ARSA) gene	GTMP	Metachromatic leukodystrophy	Orchard Therapeutics (Netherlands) BV	17/12/2020	Yes	No	
Tecartus	Brexucabtagene autoleucel	Autologous T cells transduced *ex vivo* using a retroviral vector expressing an anti-CD19 chimeric antigen receptor (CAR) comprising a murine anti-CD19 single chain variable fragment linked to CD28 co-stimulatory domain and CD3-zeta signalling domain	GTMP	Mantle cell lymphoma (MCL)	Kite Pharma EU B.V.	14/12/2020	Yes	Yes	
Skysona	Elivaldogene autotemcel	Autologous CD34^+^ cell-enriched population that contains haematopoietic stem cells transduced *ex vivo* with lentiviral vector encoding ABCD1 cDNA for human adrenoleukodystrophy protein	GTMP	Cerebral adrenoleukodystrophy	Bluebird bio (Netherlands) B.V.	16/07/2021	Yes	Yes	withdrawn Nov. 2021
Abecma	idecabtagene vicleucel	Autologous T cells transduced with lentiviral vector encoding a chimeric antigen receptor (CAR) that recognises B-cell maturation antigen	GTMP	Multiple myeloma	Bristol-Myers Squibb Pharma EEIG	18/08/2021	Yes	Yes	
Breyanzi	Lisocabtagene maraleucel	Autologous purified CD8^+^ and CD4^+^ T cells, in a defined composition, that have been separately transduced *ex vivo* using a replication incompetent lentiviral vector expressing an anti- CD19 chimeric antigen receptor (CAR) comprising a single chain variable fragment binding domain derived from a murine CD19-specific monoclonal antibody (mAb; FMC63) and a portion of the 4-1BB co-stimulatory endodomain and CD3 zeta chain signalling domains and a nonfunctional truncated epidermal growth factor receptor	GTMP	Diffuse large B-cell lymphoma (DLBCL), primary mediastinal large B-cell lymphoma (PMBCL) and follicular lymphoma grade 3B (FL3B)	Bristol-Myers Squibb Pharma EEIG	4/04/2022	No	Yes	
Carvykti	ciltacabtagene autoleucel	Autologous T cells transduced *ex vivo* using a replication incompetent lentiviral vector encoding an anti-B cell maturation antigen chimeric antigen receptor (CAR), comprising two single domain antibodies linked to a 4-1BB costimulatory domain and a CD3-zeta signaling domain	GTMP	Multiple myeloma	Janssen-Cilag International NV	25/05/2022	Yes	Yes	
Upstaza	Eladocagene exuparvovec	Non-replicating recombinant adeno-associated virus serotype 2 based vector containing the cDNA of the human dopa decarboxylase gene under the control of the cytomegalovirus immediate-early promoter	GTMP	Aromatic L amino acid decarboxylase (AADC) deficiency with a severe phenotype	PTC Therapeutics International Limited	18/07/2022	Yes	No	
Roctavian	valoctocogene roxaparvovec	Non-replicating recombinant adeno-associated virus serotype 5 based vector containing the cDNA of the B-domain deleted SQ form of human coagulation factor VIII gene under the control of a liver-specific promoter	GTMP	severe haemophilia A (congenital factor VIII deficiency)	BioMarin International Limited	24/08/2022	Yes	No	
Ebvallo	Tabelecleucel	Allogeneic Epstein-Barr virus-specific T-cell	GTMP	Epstein-Barr virus positive post-transplant lymphoproliferative disease (EBV+ PTLD)	Pierre Fabre Medicament	16/12/2022	Yes	Yes	
Hemgenix	Etranacogene dezaparvovec	Non-replicating, recombinant adeno-associated virus serotype 5 based vector containing a codon-optimised cDNA of the human coagulation Factor IX variant R338L (FIX-Padua) gene under the control of a liver-specific promoter (LP1)	GTMP	Severe and moderately severe Haemophilia B (congenital Factor IX deficiency)	CSL Behring GmbH	Opinion Dec. 2022	Yes	Yes	Commision pending

Abbreviations: ATMP, advanced therapy medicinal product; GTMP, gene therapy medicinal product; CTMP, cell therapy medicinal product; TEP, tissue engineered product; MA, Marketing authorization.

Regarding the authorization of MAAs, it is worth noting that the CAT in the European Union maintains stringent requirements for evidence of quality, safety, and efficacy before granting marketing authorization for ATMPs [7]. This rigorous approval process implies that not all MAAs submitted will be authorized, and certain products may necessitate additional data or further development to meet the necessary criteria. Furthermore, continuous monitoring and surveillance of authorized ATMPs are conducted, and the CAT may request supplementary data or undertake regulatory measures if concerns arise regarding safety or efficacy. This ongoing monitoring ensures that ATMPs continue to meet the required standards of quality, safety, and efficacy after being authorized for marketing. European Union has implemented various measures to tackle the challenges associated with the approval of ATMPs. One such measure involves the establishment of incentives to support the development of orphan drugs, including ATMPs. Orphan drugs are specifically designed to treat rare diseases that affect a relative small number of patients. Companies engaged in the development of orphan drugs, including ATMPs, can be encouraged by several incentives, such as market exclusivity, reduced fees, protocol assistance, and eligibility for EU research funding. Additionally, the EU has introduced a regulatory pathway known as “conditional marketing authorization.” This pathway enables expedited access to medicines that address unmet medical needs. Conditional approval can be granted to ATMPs and other drugs based on promising initial data. This regulation assures that the medicine meets strict EU standards for safety, quality and efficacy, and that suppporting data is still generated post-approval to complete its safety profile. The most recent example which allowed fast track authorization is the COVID-19 vaccines which were released in the EU to support the mass vaccination campaign against the corona virus. Companies that receive conditional approval are required to provide further evidence to substantiate the benefits of the medicine, thus allowing patients to access potentially life-saving treatments at an earlier stage. These measures are aimed at promoting the development of ATMPs and improving access to innovative therapies for patients affected by rare diseases or unmet medical needs. However, despite these encouragments, they may not be sufficiently effective to facilitate widespread approval and dissemination of ATMPs.

The sluggish approval process for ATMPs has various implications that warrant concern [8]. Foremost among these is the potential to leave patients with rare and complex diseases without access to life-saving treatments [9]. The delay in approvals can prolong their suffering and even lead to unnecessary fatalities, while the social economic effects of their disease lead to increased healthcare costs and less access to the labor market. Furthermore, the restricted adoption of ATMPs may impede scientific advancement in regenerative medicine and cell therapy, as they offer a new and promising means of treating diseases, as more positive examples could lead to increased interest from the scientific community to explore these alternatives. A limited number of approved ATMPs could discourage investors and researchers from pursuing this area of research, leading to a lack of progress in creating innovative cell therapies. Moreover, the high costs associated with developing ATMPs may cause companies to be wary of investing in them without a comprehensive understanding of regulatory requirements and commercial viability. The tardiness in approvals could intensify this hesitancy, leading to a decline in investment in ATMPs and a deceleration in the development of new treatments.

The challenges that ATMPs face in overcoming the “economic valley of death” after obtaining marketing authorization have raised even greater concerns [10]. In fact, out of the 25 authorized ATMPs, seven had their marketing authorization withdrawn or not renewed. It is evident that the financial commitment for earlier trials, even in academic settings, is considerably high and the cost of upgrading an ATMP manufacturing process to obtain GMP certification is substantial, often exceeding that of similar clinical-grade cell products by 2–3 times. To make the process economically sustainable, academic scientists have established collaborations with small or large companies or founded biotechnology start-ups [11]. Nonetheless, the investments needed to take an ATMP to marketing authorization are very high, not only due to the costs of running clinical trials, but also the manufacturing costs of viral vectors and cellular products, as well as the stringent standards imposed by regulatory agencies to ensure the safety and quality of these products. Furthermore, the patient population that would benefit from these therapies is often very small, ranging from several thousand for less rare diseases to a few dozen for very rare diseases. The high costs of research and development and production have resulted in companies demanding very high prices for these therapies, ranging from several hundred thousand to a few million euros per patient. This can cause lengthy negotiations or even rejection by National Health Systems unwilling to cover the costs, even for a few patients. As a result, some efficacious and approved products, such as Skysona for adrenoleukodystrophy and Zynteglo for beta-thalassemia, have been withdrawn from the European market, leaving patients who might have benefited from these life-saving therapies without access to them. Secondly, companies may decide to drop an approved ATMP, even when it is approved and when a reimbursement policy has been negotiated, simply due to insufficient economic return [12]. This is particularly true for rare diseases where the very low number of patients poses significant challenges. For instance, Glybera, an approved gene therapy product for Type I hyperlipoproteinemia, withdrew after treating only one patient. Similarly, Strimvelis®, the first *ex vivo* gene therapy product approved in Europe, was passed on to Orchard Therapeutics after being created by GSK and the San Raffaele Telethon Institute of Gene Therapy in Milan. While the therapy proved effective in treating some patients, Orchard Therapeutics recently pulled it from the market due to commercial considerations. Finally, Valline Holding Srl made the decision to cease financial backing to Holostem, the company responsible for creating Holoclar®, the first stem cell-derived ATMP approved in Europe.

Developing ATMPs for rare diseases is crucial to the advancement of medical innovation and technology transfer [13]. The development of these therapies requires significant investments in research, development, infrastructure, and regulatory frameworks, which can be leveraged to develop treatments for more common diseases. This approach encourages collaborations between academic institutions, industry partners, and regulatory agencies, stimulating innovation and accelerating the translation of research into clinical applications. In addition, the development of ATMPs for rare diseases can create an ecosystem that supports innovation and technology transfer. Researchers and developers can use these rare diseases as a platform to refine cutting-edge technologies, such as gene editing and stem cell therapies, which can then be applied to other disease areas with similar genetic and cellular foundations, such as cancer or neurodegenerative diseases. Moreover, developing ATMPs for rare diseases can spur investment in related fields, including manufacturing and supply chain logistics, helping to lower the production costs and improve the scalability of ATMPs, ultimately making them more accessible to patients worldwide.

ATMPs are extremely relevant to the field of transplantation as they offer a promising way to address many of the challenges associated with organ and tissue transplantation [14, 15]. One of the main challenges of transplantation is rejection, which occurs when the recipient’s immune system recognizes the transplanted organ or tissue as foreign and attacks it. Immunosuppressive therapy is currently used to prevent rejection, but this can have significant side effects and long-term complications, including increased susceptibility to infections and cancer. ATMPs offer a potential solution to this problem by modifying the recipient’s immune system to accept the transplanted organ or tissue as “self.” For example, chimeric antigen receptor (CAR) T cell therapy involves genetically engineering the patient’s own immune cells to target and destroy cancer cells [16]. This approach has shown promise in treating post-transplant lymphoproliferative disorders, which are a common complication of solid organ transplantation. Another ATMP approach is the use of regulatory T cells (Tregs), which are a subset of immune cells that play a key role in immune tolerance [17]. Treg therapy is being developed to induce immune tolerance and reduce the need for immunosuppressive therapy, which could improve patient outcomes and reduce the risk of complications. ATMPs are also being used to address other challenges associated with transplantation, such as the limited availability of donor organs and tissues. Tissue engineering is one approach that involves using biodegradable scaffolds and cells to create functional replacements for damaged or diseased tissues (cartilage, bone, skin, vessels, islet, etc.). Xenotransplantation is another approach that involves transplanting organs or tissues from one species to another. ATMPs such as gene editing and immune cell therapies are being developed to overcome the immunological barriers associated with xenotransplantation and make it a viable option for treating organ failure.

The field of transplantation is at a critical juncture, as there is an urgent need to address the challenges associated with organ and tissue transplantation. ATMPs offer a promising way to achieve this goal, but their development and access to academic research must be sustained and expanded to fully realize their potential [18]. The transplant community has a critical role to play in sustaining the ATMP field, as they are uniquely positioned to identify the unmet needs and opportunities for innovation in transplantation. This includes advocating for increased funding for ATMP research and development, as well as promoting collaborations between academic researchers, industry partners, and regulatory agencies to accelerate the translation of promising ATMP therapies to the clinic. In addition, the transplant community can support the development and adoption of innovative approaches to transplant surgery, such as *ex vivo* organ perfusion, which has been shown to improve the quality of donor organs and increase the number of viable organs available for transplantation. By embracing new technologies and approaches to transplantation, the transplant community can create a more supportive environment for the development and adoption of ATMPs. Moreover, it is essential that the transplant community engage in ongoing education and training on the latest advances in ATMPs, including their potential clinical applications, regulatory considerations, and ethical implications. This will ensure that transplant clinicians and researchers are equipped with the knowledge and skills needed to effectively translate and apply ATMPs in the clinical setting. Ultimately, the success of the ATMP field in transplantation will depend on the sustained commitment and collaboration of the transplant community. By working together to overcome the regulatory and funding challenges associated with ATMP development and access, the transplant community can help to ensure that patients in need of organ and tissue transplantation have access to the most innovative and effective therapies available. The timely and invaluable action of launching a task force by the European Society of Organ Transplantation (ESOT) to address ATM field issues in Europe demonstrates a recognition of the pressing challenges faced in the academic institution. It highlights a strong commitment to finding effective solutions. By assembling experts and stakeholders, the task force can capitalize on their collective knowledge and expertise to address crucial issues, fostering innovation, efficiency, and safety in European member states.

More specifically, to overcome the bottleneck in the development and access to ATMPs for academic research in the field of transplantation, several strategies can be implemented [19, 20]:

i) Streamlining regulatory processes. One of the major barriers to the development and access of ATMPs is the complex and lengthy regulatory approval process. To overcome this bottleneck, regulatory agencies can work to streamline their processes and reduce the time and cost of approval, while still ensuring the safety and efficacy of these therapies [21]. The EMA/CAT definition of ATMPS warrants reconsideration. Is it appropriate to regulate minimally modified cell therapy products, such as Stromal Vascular Fraction (SVF) differently based on non-homologous (ATMP) versus homologous therapy (simple cell therapy)? For instance, comparing SVF for plastic surgery (simple cell therapy) and SVF for scleroderma (ATMP), should not the classification be determined by the manufacturing process rather than the clinical end use? Could production facilities conduct risk assessments as evidence of manufacturing process quality?
ii) Increasing funding for ATMP research. The development of ATMPs requires significant investment in research and development. A challenge arises from the fact that public funding typically does not support these endeavors. National science funding primarily prioritizes the creation of new knowledge, focusing on academic research rather than the establishment of clinical trials or conducting safety studies, activities classified as TRL4 and higher in the EU. Consequently, scaling up an ATMP becomes unfeasible due to insufficient funding. To support this, funding agencies can increase their investment in ATMP research, with a focus on academic research and development.
iii) Promoting collaborations and partnerships. Collaboration between academic researchers, industry partners, and regulatory agencies is critical to the development and translation of ATMPs. To facilitate this, there is a need for increased support for partnerships and collaborations, including funding, infrastructure, and regulatory support.
iv) Establishing/Supporting Pre-ATMP Facilities. The development of pre-ATMP facilities is crucial for improving the efficiency and success rate of ATMP projects. It provides researchers with a valuable platform to thoroughly test their products, ensuring compatibility, safety, and efficacy before committing significant resources to full-scale GMP production. By avoiding potential pitfalls related to raw material and starting material selection early on, researchers can streamline the translation of ATMPs into clinical trials, fostering a more effective and efficient development pathway.
v) Establishing centralized ATMP facilities. The successful development and production of ATMPs rely on specialized facilities and expertise. To address the limited availability of these resources, the establishment of centralized ATMP facilities can provide academic researchers with accessible infrastructure and regulatory affairs expertise. This point is crucial, as it emphasizes the importance of not only having such facilities but also ensuring their affordability and having the necessary regulatory affairs expertise readily available. Having a cleanroom facility alone is insufficient for achieving clinical translation. The progress towards this goal can greatly benefit from experts who possess knowledge not only about regulatory hurdles but also about establishing effective quality management systems, training personnel in Good Manufacturing Practices (GMP), defining release criteria, and navigating other approval requirements. Typically, one option is to rent a cleanroom facility and seek the assistance of consultants for regulatory affairs. Additionally, safety studies are often outsourced to third-party organizations with ISO certification. However, this approach tends to be expensive, and obtaining research funding for these activities can be extremely challenging. This is because they extend beyond the scope of academic work and require substantial financial investments. Consequently, a gap is created presenting significant obstacles in terms of both financial resources and expertise for research groups interested in pursuing ATMP development. It would be beneficial if centralized facilities could offer a cost-effective combination of services specifically designed for academic researchers.
vi) Enhancing the efficiency and accessibility of the “Hospital Exemption” (HE) approval pathway. The HE pathway, a regulatory framework outlined in European Regulation No 1394/2007, provides a means for manufacturing and utilizing ATMPs outside the standard centralized marketing authorization pathway, subject to specific conditions. In February 2021, ARI-0001 (CART19-BE-01), an ATMP designed to target CD19^+^ B-cell malignancies, achieved a significant milestone [22]. The Spanish Agency of Medicines and Medical Devices (AEMPS) authorized its use under the HE pathway for treating adult patients over 25 years old with relapsed/refractory acute lymphoblastic leukemia. This achievement is remarkable as ARI-0001 becomes the first CAR-T therapy to receive approval from a governmental drug agency outside the central marketing authorization pathway. However, it is important to acknowledge the emergence of divergent interpretations and variations in HE implementation across countries and within industrial or academic organizations. These differences [8, 23–26] underline the need for greater harmonization of HE rules. While it is widely agreed that the HE pathway should not be exploited to bypass established procedures for marketing authorization and clinical trials in Europe, different viewpoints exist regarding the reasons for utilizing this pathway. Academic organizations emphasize the need to ensure uninterrupted patient treatment during clinical development, reduce costs, provide therapeutic options for individuals ineligible for clinical studies, accommodate early stages of product development with rapid manufacturing and advancements, and enable access to ATMPs with limited commercial viability that may not progress towards marketing authorization. Conversely, industrial organizations primarily raise concerns about the potential risks associated with establishing a dual-tier system with varying regulatory standards. Numerous challenges are associated with the HE pathway that necessitate attention: a) the lack of harmonization of HE rules among EU Member States, b) the necessity to enhance flexibility and efficiency in the regulatory process for HE-ATMPs, where even minor product modifications are regarded as “new products,” c) the substantial requirement of human, logistic, and financial resources, which pose barriers for both public facilities and private investors, particularly small and medium enterprises, d) ensuring access to HE-ATMPs for patients treated in hospitals other than the one involved in product development, and e) facilitating technology transfer and knowledge sharing to promote access to these therapies in hospitals within or beyond the Member State.
vii) Engaging with patients and patient advocacy groups. Patients and patient advocacy groups should have an important role in the clinical development and translational process of ATMPs. Early exploration and engagement of patient perspectives are essential to understand and address the barriers and facilitating factors that may affect the uptake and impact of ATMPS on patient communities. Empirical research on patient perspectives is therefore important in order to ensure responsible clinical translation of ATMPs.

Finally, accessibility implies not only availability, but also affordability [27]. Given the expected high prices of ATMPs, there are concerns about equitable distribution of ATMPs. However, especially in countries in which ATMP facilities and trained staff are lacking, these treatments may not become accessible to patients who may need them most. To provide equitable access to ATMPs across different regions and communities, investments must be made in robust supply chains and knowledge sharing. Even in less resource-constrained settings, strategies for fair pricing will be required, as well as adequate reimbursement policies and the provision of support programs to alleviate financial burdens on individual patients. Particular attention should be paid to individuals with rare diseases, as they often face significant challenges in accessing effective treatments. Achieving fair distribution of ATMPs entails addressing patients’ needs, including by raising awareness, improving early diagnosis, and establishing support networks. By doing so, we can ensure that patients with unmet medical needs have equitable opportunities to benefit from ATMPs. Equity in ATMPs extends to the realm of clinical trials as well. It is essential to ensure the inclusion of diverse populations, including historically underrepresented groups, in research studies. This inclusive approach enables a comprehensive understanding of the benefits and risks of ATMPs across various patient populations, thereby avoiding potential biases and ensuring equitable access to the benefits of research. Lastly, global disparities must be addressed to achieve equity in access to ATMPs. Efforts should be made to bridge gaps between different countries and regions, allowing individuals worldwide to benefit from these therapies. This can be accomplished through international collaboration, regulatory harmonization, and the transfer of knowledge and technology. By placing fair distribution of ATMPs at the center of our ethical considerations, we can work collectively to establish a healthcare landscape where all individuals, regardless of their socio-economic status, disease rarity, geographic location, or background, have equitable access to the transformative potential of ATMPs.

To claim for a new time, it is important to advocate for changes at the policy level that support the development and access to ATMPs for academic research [28]. This includes advocating for increased funding, streamlined regulatory processes, engagement of patients and patients advocacies and collaboration between academic researchers, industry partners, and regulatory agencies [29]. By working together to overcome the bottleneck in the development and access of ATMPs, we can create a more supportive environment for innovation in the field of transplantation and help to improve patient outcomes.

## Data Availability

Publicly available datasets were analyzed in this study. This data can be found here: [2].
